# Formal Elements of Art Products Indicate Aspects of Mental Health

**DOI:** 10.3389/fpsyg.2020.572700

**Published:** 2020-09-25

**Authors:** Ingrid Pénzes, Susan van Hooren, Ditty Dokter, Giel Hutschemaekers

**Affiliations:** ^1^Department of Arts Therapies, Faculty of Health Care, Zuyd University of Applied Sciences, Heerlen, Netherlands; ^2^KenVaK Research Centre for the Arts Therapies and Psychomotricity, Heerlen, Netherlands; ^3^Faculty of Psychology, Open University of the Netherlands, Heerlen, Netherlands; ^4^MA Music Therapy and Drama Therapy, Cambridge School of Creative Industries, Anglia Ruskin University, Cambridge, United Kingdom; ^5^Master Dance Therapy, Codarts University, Rotterdam, Netherlands; ^6^Faculty of Social Sciences, Radboud University, Nijmegen, Netherlands; ^7^Pro Persona Mental Health Care, Nijmegen, Netherlands

**Keywords:** art therapy observation, assessment, formal elements, art product, adult mental health

## Abstract

Formal elements are often used in art therapy assessment. The assumption is that formal elements are observable aspects of the art product that allow reliable and valid assessment of clients’ mental health. Most of the existing art therapy assessment instruments are based on clinical expertise. Therefore, it is not clear to what degree these instruments are restricted to formal elements. Other aspects might also be included, such as clinical expertise of the therapist. This raises the question of whether and how formal elements as observable aspects of the art product are related to clients’ mental health. To answer this question, four studies are presented that look at: (1) a meta-theoretical description of formal elements; (2) operationalization of these formal elements so they can be analyzed reliably in clients’ art products; (3) establishment of reliable and clinically relevant formal elements; (4) the relationship between formal elements and adult clients’ mental health. Results show that the combination of the formal elements “movement,” “dynamic,” and “contour” are significantly interrelated and related to clients’ mental health, i.e., psychopathology, psychological flexibility, experiential avoidance, and adaptability. These findings give insight in the diagnostic value of art products and how they may add to clients’ verbal expression and indicate their potential to benefit from therapy.

## Introduction

Art therapy is an experiential treatment in which the art form is therapeutically applied to initiate experiences that allow processes of change, development, stabilization or acceptance that support emotional, behavioral, cognitive, social, neurological or physical aspect of functioning (American Art Therapy Association [AATA], 2020; British Association of Art Therapists [BAAT], 2020). The art form – the use of art materials in an art-making process resulting in an art product– is specific and unique for art therapy treatment and for art therapy observation and assessment. Art therapy approaches fundamentally assume that a clients’ mental health is expressed in the art form (Hinz, 2009, 2015, 2020; Haeyen, 2018; Abbing et al., 2019).

Art therapy observation and assessment is considered to be a key to estimate what the client’s problem is, to determine whether a client may benefit from art therapy, to formulate treatment goals and to apply the appropriate art interventions (Hinz, 2009, 2020; Gilroy et al., 2012; Malchiodi, 2012). Several art therapy perspectives that draw from diverse approaches such as psychodynamic, humanistic, behavioral, developmental and integrative approaches (Rubin, 2001,2005; Jones, 2005; Van Lith, 2016), have different views and theories on how exactly the art form relates to mental health. Most art therapists adhere clinical value to the art products of their clients. They recognize in art products signs of their clients’ mental health, as well as cues on how to treat, i.e., what therapeutic goals and interventions should be chosen (indication).

Testimonies of the clinical value of the art product can be traced back to the beginning of the 20th century (Prinzhorn, 1919; Plokker, 1963). Already before WW II all kind of projective drawing tests were developed pretending to provide insight in unconscious psychological conflicts (Murray, 1943; Buck, 1948; Naumburg, 2001; Simon, 2001; Ulman and Bernard, 2001). From a psychometric perspective, these tests lacked reliability and validity; it appeared impossible to unequivocally score an art product in terms of content (McNiff, 1998; Betts, 2006). The focus subsequently shifted from symbolic content to the formal elements of art products, such as “line,” “color,” and “movement” as these were considered to be more objective (Gantt, 2001, 2004; Thomas and Cody, 2012). The underlying assumption is that formal elements in an art product represent the way clients interact with art materials in order to express themselves and that they are related to clients’ mental health (Gantt and Tabone, 1998; Gantt, 2001, 2004; Hinz, 2009, 2015; Gilroy et al., 2012; Nan and Hinz, 2012; Pénzes et al., 2014, 2015). Several art therapy assessment instruments have been developed to measure these formal elements and relate them to clients’ mental health problems (e.g., Cohen,1986/1994; Cohen et al., 1986; Hacking, 1999; Elbing and Hacking, 2001; Stuhler-Bauer and Elbing, 2003). However, these instruments have been subject to serious psychometric critique regarding their reliability and validity. It has been argued that the existing instruments use poor rating methods (Nan and Hinz, 2012) and inappropriate statistical procedures for measuring inter-rater reliability (Hacking, 1999; Eitel et al., 2008; cited in Betts, 2006; Schoch et al., 2017).

The existing instruments also allow, and even need, subjective interpretation based on art therapists’ own clinical experiences with different client groups and different training orientations (Betts, 2006; Eitel et al., 2008; Schoch et al., 2017). This introduces all manner of interpretations, which can negatively affect the reliability. We still do not know to what degree formal elements as the unambiguous observable aspects of an art product represent the mental health problems of clients or how the clients cope with these problems. Critics of existing instruments also refer to the wide range of opinions regarding how formal elements should be interpreted in terms of mental health (Pénzes et al., 2018). Due to these shortcomings we still do not know whether or not art products can be used for diagnostic and indicative purposes. We know that the content of the art product cannot be measured in a reliable and valid way, but it is still possible that formal elements as the unambiguous observable aspects of an art product represent the mental health problems of clients. The aims of this study are to define formal elements that can be operationalized and analyzed reliably and are clinically relevant so their relationship with mental health can be tested.

**FIGURE 1:**
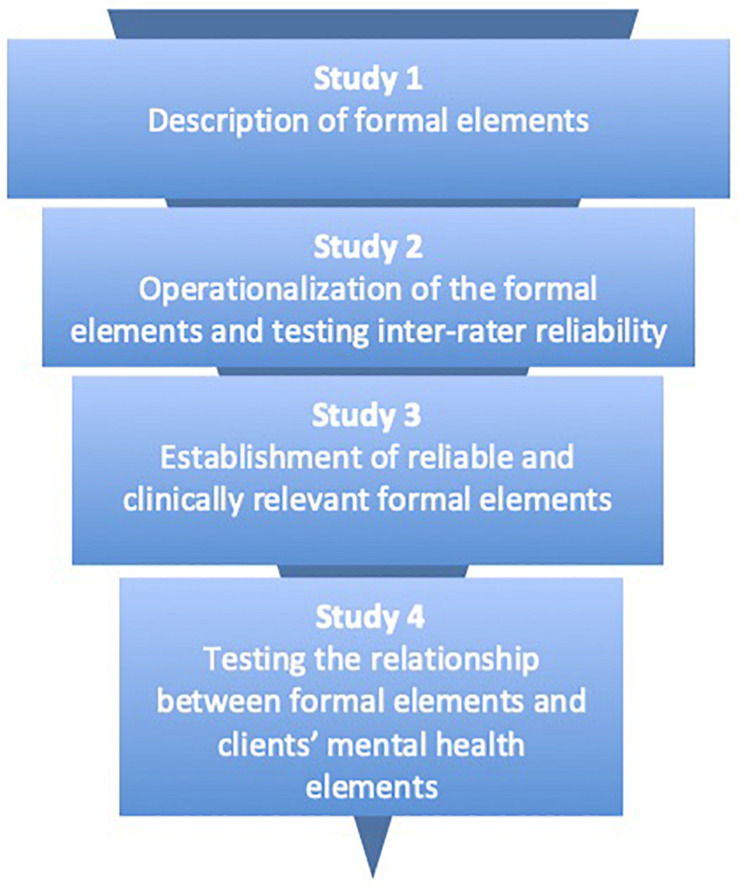
Brief descriptions of the four studies presented.

In this article, we report four studies that look at: (1) a taxonomy of formal elements; (2) an operationalization of these formal elements to analyze them reliably in clients’ art products; (3) establishment of reliable and clinically relevant formal elements; (4) the relationship between formal elements and adult clients’ mental health (see Figure 1). We will make use of qualitative as well as quantitative research designs.

## Study 1: Toward a Taxonomy of Formal Elements

In art therapy literature and clinical practice, a large number and diverse formal elements were described as clinically relevant. Even if considered as sign of the richness embedded in the clinical practice of art therapy, this huge number did not help the growth of sound empirical studies on formal elements. Aim of this first study was to clarify this complexity by returning to art theories on formal elements. Formal analysis of art refers to the observable pictorial qualities of art and is considered to be the most objective way of analyzing art products (Acton, 2009; De Visser, 2010).

### Method

To describe the formal elements, art theory literature was reviewed and experts in the field consulted to gain meta-theoretical descriptions of formal elements. Art theories that presented a complete systematic “form analysis” of visual art were included, whereas theories that concerned only one category of form, such as “line,” art history, art criticism, art philosophy, neurology and art perception, and/or aesthetics were excluded. As art theories cannot be found in scientific databases, three other sources were used. First, existing textbooks on art theory as found on the Internet and the university’s library. Additionally, six experts in the field of art theory, specifically art analysis, were consulted by email for advice regarding which additional art theories to include. These experts consisted of teachers/researchers from the Netherlands, Germany, Israel, and Finland. These experts were chosen based on their expertise in the field of art theory, specifically form analysis, and/or in using formal elements in art therapy. Also, bibliographies of art therapy literature regarding formal elements in assessment were searched.

All included art theories were incorporated into a comparative content analysis to see which formal elements were included and how they were described. We restricted our search to those formal elements that could be appropriately be described in two-dimensional art because this is mainly used in art therapy assessment (e.g., Moon, 2010; Pénzes et al., 2014). We began with four theories that incorporated a complete systematic “form analysis” of visual art: Acton (2009), the KPC model (Gerrits, 2018), the ILO UvA model (Van der Kamp et al., 2012); and Tate Modern (Gale, 2016; Wilson and Lack, 2016). Additionally, the consultation of the experts led us to the theories of De Visser (2010), Taylor (2014), Huntsman (2016), and Boermans and van der Borght (2017). Eight theories were included in further comparative content analysis: the ILO UvA model (Van der Kamp et al., 2012), Acton (2009), the KPC model (Gerrits, 2018), Tate Modern (Gale, 2016; Wilson and Lack, 2016), De Visser (2010), Taylor (2014), Huntsman (2016), and Boermans and van der Borght (2017). We extracted the formal elements included in all theories and their descriptions were compared for similarities.

### Results

The comparative content analysis showed that all incorporated art theories divided the formal elements into categories. We recognized six main categories: shape, composition, color, space, surface quality and light. Based on the comparative content analysis we found agreement on 17 formal elements across these categories: (1) “**shape**,” including “geometry” and “contour”; (2) “**composition**,” including “symmetry,” “rhythm,” “line quality,” “linearity,” “movement,” and “dynamic”; (3) “**color,**” including “number of colors,” “color saturation,” “color brightness,” “hue,” and “mixture of color”; (4) “**space**,” including “filled area,” “used space,” and “suggestion of space,” (5) “**surface quality**,” including “texture” (see Table 1), and (6) “**light**,” including “floodlight,” “light source,” and “clair-obscure.” Formal elements of category 6 mainly concern photography or art products with realistic styles as in “baroque” and “romanticism.” Because these are rarely seen in art therapy, we did not include this category in our model.

**TABLE 1:** The five categories and the seventeen formal elements.

1. Shape
**Geometry**
Geometry refers to geometric shapes, such as rectangles, circles, and triangles. The opposite of geometric is organic.
**Contour**
Contour refers to the sharp edges, outlines, and borders of and between separate parts of the art product. Contour is formed by the use of sharp lines or distinct borders. Shapes are completely accentuated by lines and/or have distinct borders as if they were “cut out.” The opposite of contour is pictorial, diffuse artwork.

**2. Composition**

**Symmetry**
Symmetry is formed by an optical vertical axis of the middle of the artwork. The left and right half of the art product are mirrored and are equal with regard to color and/or shape, line, or figure. The opposite of a symmetric composition is a chaotic overall composition.
**Rhythm**
Rhythm refers to the regular return of lines, shapes, or colors. It is a repeated pattern that can be predicted once it is recognized.
**Line quality**
The quality of a line is strong when it is serried, straight, sharp, and fluent. The opposite is a line that is broken, sketchy, wavered, crumbled, and cautious. To judge line quality, the quality of the lines present is observed rather than the number of lines.
**Linearity**
Linearity refers to the extent to which an artwork consists of lines. A line is a stripe not broader than the tip of the used tool (graphic art material) or the brush. Lines can form shapes. The opposite is a pictorial artwork that lacks lines.
**Movement**
Movement consists of a combination of elements, such as diagonals and overlap. Movement varies in amount.
**Dynamic** Dynamic refers to the tension within the artwork. Tension is created when the artwork seems to deny the frame. This is called “a-tectonic.” The opposite is an artwork that shows no tension with the frame.

**3. Color**

**Number of colors**
The number of distinct, used colors. An artwork can be very colorful and have many colors. The opposite is an artwork that consists of a limited number of colors.
**Color saturation**
The extent to which a color is saturated. The more saturated the color is, the less paper/canvas, etc., is apparent.
**Color brightness**
Bright colors sparkle and are pure and fresh, such as the green of grass, the yellow of the sun, and the blue of the sky. The color brightness of an artwork increases with the number of bright colors used. Colors that are mixed with water, white, or black (dark or pastel colors) are not bright.
**Hue**
Hue refers to gradience from light to dark tones of one color or grays (not different “families of color” such as greens, blues, cold or warm colors).
**Mixture of color**
The amount of which colors are visibly mixed. The opposite is an art product in which the colors are not mixed and applied separately.

**4. Space**

**Filled area**
Filled area refers to the “worked” space on the paper or canvas and the ratio between used and unused space.
**Used space**
Used space refers to the space on the paper/canvas, etc., that is used.
**Suggestion of space**
Space can be suggested by several techniques, such as overlap, shadow, line perspective, atmospheric perspective, and foreground and background. The suggestion of depth is created. The opposite refers to a “flat” artwork without any suggestion of space.

**5. Surface quality**

**Texture**
Texture refers to the tactile surface of the artwork. This does not depend on the used paper or canvas but can depend on the art material used and the way it is applied.

All art theories conceptualized formal elements as continua with dominant presence of absence in the art product as polarities. Also, all the theories mentioned that formal elements can reinforce or weaken each other.

In conclusion, among a large number of elements we found agreement about seventeen formal elements described in terms of continua. The next step was to analyze whether it was possible to describe and observe them reliably in art products of adult clients. Therefore, a second study was conducted.

## Study 2: Operationalization and Inter-Rater Reliability

In this study, we investigated whether it was possible to construct reliable formal elements in the art products of adult clients in art therapy. The formal elements that emerged from study 1 were operationalized and tested for inter-rater reliability.

### Method

**FIGURE 2:**
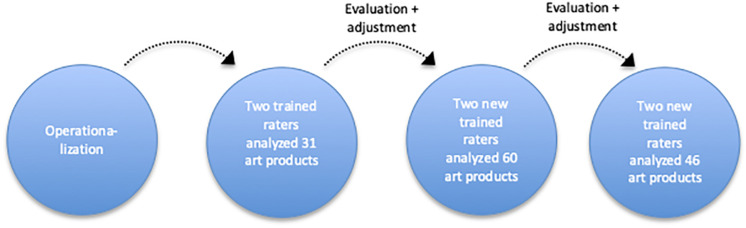
Chart of the procedure followed in study 2.

The formal elements that emerged in study 1 were operationalized in three test rounds (see Figure 2). This was peer debriefed by two art therapy teachers. Each round was evaluated with the raters and researcher. In the first round, two trained raters independently analyzed 31 art products made by nine adult clients in one mental health setting. The operationalization of the formal elements was adjusted based on the evaluations and tested in a second round where two new trained raters independently rated 60 art products from 25 clients in six mental health settings. Their ratings were tested for inter-rater reliability and evaluated. The operationalization of some of the formal elements was adjusted based on the evaluation. Two new trained raters then independently rated 46 art products from 46 clients in nine mental health settings.

In total, 137 art products from 80 clients were included in this study: 31 art products from 9 clients in the first round, 60 art products from 25 clients in the following round, and 46 art products from 46 clients in the final round. Client inclusion criteria included being aged between 18 and 65 years, being able to read and speak Dutch, and having no or minimal experience in making art and art therapy. Clients with psychotic disorders or in manic episodes were excluded. In line with the Declaration of Helsinki, informed consent was obtained from all clients. The art products in the first two rounds were made as part of regular art therapy assessment, meaning they were of diverse sizes and styles and used diverse two-dimensional art materials like pastel, pencil, or ink. Some of the clients were instructed while making the products, while other art products were made based on the clients’ own choices. The art products in the final round were made with standardized semi-structured instruction that consisted of painting a landscape with acrylic paint on paper of 50 × 40 cm in a 45-min session. All clients received the same color palette, the same brushes and pencils, and the same instructions from their therapists. The therapists were given paper and video instructions and took part in a telephone meeting to confirm that all their clients would receive the same instructions.

The sampled clients (52 female, 28 male) started art therapy in 16 different settings for adult mental health care ranging in age from 19 to 60 (*M* = 39). They all just started art therapy, just after a psychologist gave a preliminary DSM-diagnosis. The sample was very heterogenic with regard to the preliminary diagnoses. Most clients (39%) were diagnosed with two or three diagnoses. Personality disorder (72%) and mood disorder (56%) were diagnosed most frequently.

In the first round, the formal elements were operationalized categorically and analyzed for percentage of agreement. Finally, the formal elements were operationalized on a five-point Likert scale and tested for inter-rater reliability using Cohen’s kappa analysis. All analyses were conducted using SPSS version 25.

### Results

#### Operationalization

**FIGURE 3:**
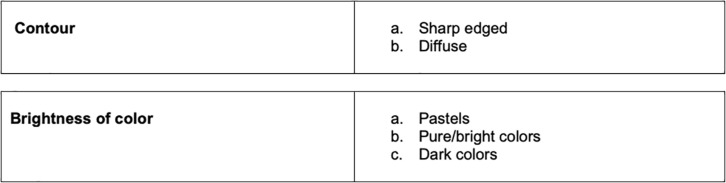
Examples of categorical operationalization.

Initially, the formal elements were operationalized by categories. Some of the formal elements consisted of two categories, while others consisted of three (see Figure 3 for examples).

**FIGURE 4:**
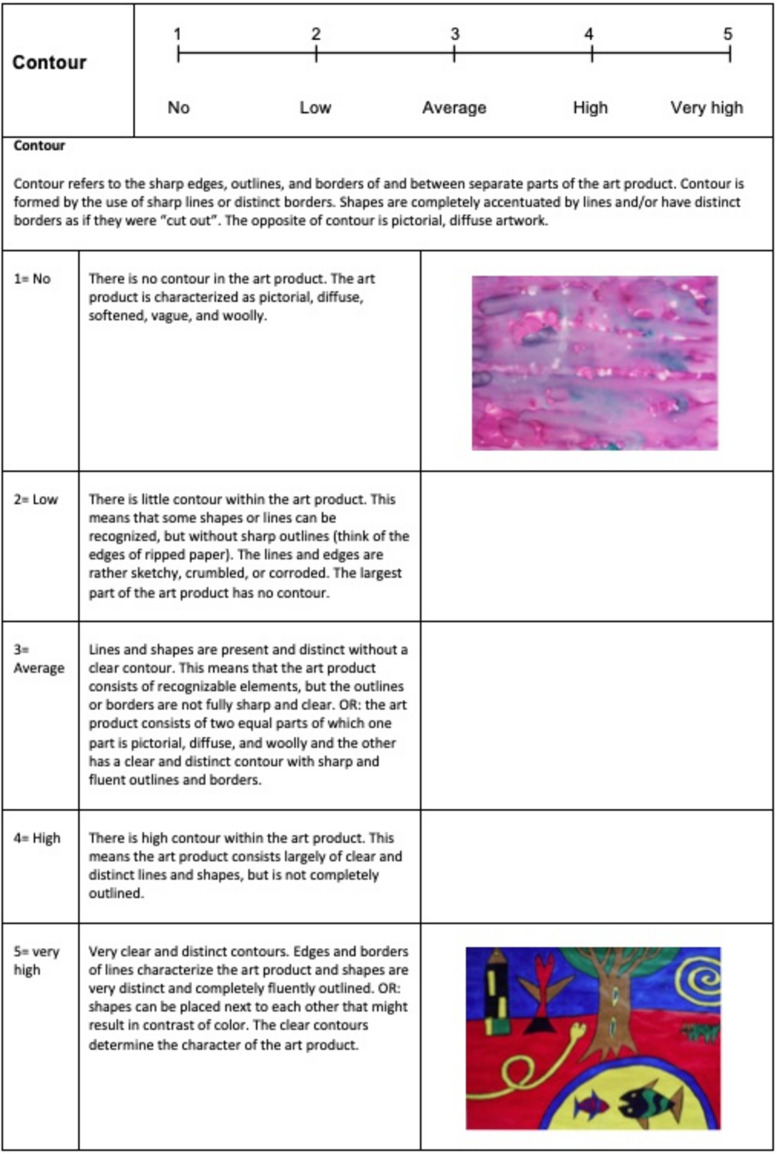
Example of operationalization of the formal element “contour,” (The appropriate permissions have been obtained from the copyright holders of this work).

The overall inter-rater agreement was >88%, but despite this high agreement, the raters found it difficult to rank the formal elements with different numbers of categories. They also found it difficult to rate the art products based on the written definitions of the formal elements and suggested adding visual illustrations. The operationalization was adjusted and all the formal elements were operationalized into five-point Likert scales (see Figure 4), with 1 representing no or minimal presence of the element and 5 representing a very high presence of the element. For each formal element, example images of art products were provided for both ends of the scale.

#### Inter-Rater Reliability

In round two, the inter-rater reliability for “rhythm” and “movement” was fair (κ = 0.34 and κ = 0.29, respectively), while the reliability for “contour” (κ = 0.57), “color saturation” (κ = 0.43), and “mixture of colors” (κ = 0.47) was moderate. Reliability for “suggestion of space” (κ = 0.65), “number of colors” (κ = 0.65), “color brightness” (κ = 0.64), “symmetry” (κ = 0.68), “linearity” (κ = 0.74), “line quality” (κ = 0.64), “dynamic” (κ = 0.71), and “texture” (κ = 0.65) was substantial, while reliability for “filled area” (κ = 0.82), “used space” (κ = 0.86), “geometry” (κ = 0.83), and “hue” (κ = 0.83) was almost perfect (Viera and Garrett, 2005).

The evaluation of round two highlighted three drawbacks of rating. First, the definitions of scores 3, 4, and 5 for “number of colors” overlapped. Second, raters found the definition of the item “rhythm” too complex and the definition of “movement” was found to be unclear and incomplete. This could explain the relatively low reliability for these items. All other definitions were clear and the art product examples were found to be illustrative. Third, it was questioned whether the art materials might influence the ratings of the formal elements.

Based on these drawbacks, the definitions of “number of colors,” “rhythm,” and “movement” were adjusted and tested for inter-rater reliability in a third and final round. The inter-rater reliability varied between moderate and substantial for all items except “texture” which had poor inter-rater reliability (see Table 2). The inter-rater reliability for the adjusted formal elements “number of colors,” “rhythm,” and “movement” increased in the third round. The inter-rater reliability of all other formal elements remained similar. Only the inter-rater reliability for “texture” decreased significantly, therefore this item was not included in the subsequent studies. In conclusion, 16 formal elements could be operationalized reliably. The next step was to examine which of these 16 formal elements were also clinically relevant. Therefore, a third study was conducted.

**TABLE 2:** Final inter-rater reliability scores for each formal element.

Formal elements	κ
Filled area	0.64**
Used space	0.79**
Suggestion of space	0.75**
Geometry	0.86**
Contour	0.56**
Number of colors	0.83**
Color saturation	0.76**
Color brightness	0.80**
Hue	0.71**
Mixture of color	0.68**
Symmetry	0.61**
Rhythm	0.41**
Linearity	0.56**
Line quality	0.63**
Movement	0.50**
Dynamic	0.65**
Texture	−0.012

**p < 0.001.

## Study 3: Clinically Relevant Formal Elements

We were able to operationalize most formal elements reliably, however, it was still unknown which of these formal elements were clinically relevant. Especially as the art therapy literature was inconsistent in presenting tools or suggestions directed toward the clinical use of formal elements in art therapy observation and assessment (Schoch et al., 2017; Pénzes et al., 2018). In a previous study (Pénzes et al., 2018), using Constructivist Grounded Theory, eight art therapists with diverse clinical backgrounds, training orientations, and nationalities were interviewed in-depth to identify which formal elements they observed in their clinical practice, how they described mental health and how they associated formal elements with mental health. Findings of this study revealed that seven formal elements appeared to be clinically relevant. Moreover, the art therapists in this study also observed a combination of the formal elements “movement,” “dynamic,” “contour,” and “repetition” which they used to construct the overall structure and variation of the art product (i.e., primary formal elements). And in addition “mixture of color,” “figuration,” and “color saturation” as weakening or enhancing cues for this structure (i.e., secondary formal elements).

In this study we tried to relate these seven formal elements to the reliably operationalized formal elements in study 2 above. Therefore, in this study, we compared the formal elements that were operationalized reliably in study 2 with the formal elements art therapists found to be clinically relevant from the above mentioned previous study (Pénzes et al., 2018).

### Method

A panel consisting of three art therapy teachers compared the formal elements identified by art therapists in the prior study of Pénzes et al. (2018) to the formal elements that were operationalized reliably in study 2. These formal elements, including their descriptions from art theories and art therapists, were incorporated into a table. “Figuration” was excluded, as art theories stated that this element could not be analyzed according to the level of its presence in an art product. Instead it is a categorical item that refers to the style of the art product as realistic, figurative, or abstract.

First, the comparison concerned which formal elements were identified by art therapist and art theory. Second, the descriptions of these formal elements were compared for similarities. Each expert independently compared the formal elements on a scale ranging from “0 = no similarities,” “1 = some resemblance,” and “2 = similar or almost similar.”

### Results

The panel agreed that the formal elements “contour,” “movement,” “dynamic,” and “mixture of color” were similarly described (see Table 3). “Color saturation” was described differently because the art therapists had merged “color saturation” and “texture.” The way the art therapists described “repetition” showed overlap with the formal elements “symmetry” and “rhythm” in art theory.

**TABLE 3:** Comparison of clinically relevant, objective and reliable formal elements.

	Clinically relevant formal elements according to art therapists	Formal elements operationalized in art theories	Agreement of experts
Primary	**Movement** Movement refers to the amount, character, and direction of the movement. Movement becomes visible by the brush marks.	**Movement** Movement consists of a combination of elements, such as diagonals and overlap. Movement varies in amount.	All experts gave a rating of “2”
	**Dynamic** Dynamic refers to tension (tectonic) within the art product. Dynamic varies from static, restrained, and calm to fast, turbulent, energetic, and forceful. It refers to the vitality of the movement made.	**Dynamic** Dynamic refers to the tension within the artwork. This tension is created when the artwork seems to deny the boundaries of the frame. This is called “a-tectonic.” The opposite is an artwork that shows no tension with the frame.	All experts gave a rating of “2”
	**Contour** Contour refers to the delimitation that emerges when shapes are outlined or are placed directly next to each other. This leads to rigid and sharp distinctions.	**Contour** Contour refers to the sharp edges, outlines, and borders of and between separate parts of the art product. Contour is formed by the use of sharp lines or distinct borders. Shapes are completely accentuated by lines and/or have distinct borders as if they were “cut out.” The opposite of contour is pictorial, diffuse artwork.	All experts gave a rating of “2”
	**Repetition** Repetition refers to the return of one or more formal elements in a pattern. A lot of repetition leads to symmetry. Rhythm refers to the repetition of movement.	**Symmetry** Symmetry is formed by an optical vertical axis of the middle of the artwork. The left and right half of the art product are mirrored and are equal regarding color and/or shape, line, or figure. The opposite of a symmetric composition is a chaotic overall composition. **Rhythm** Rhythm refers to the regular return of lines, shapes, or colors. It is a repeated pattern that can be predicted once it is recognized.	All experts gave a rating of “1”
Secondary	**Mixture of color** Mixture of color refers to the extent to which the colors are mixed within the art product.	**Mixture of color** The amount of which colors are visibly mixed. The opposite is an art product in which the colors are not mixed and applied separately.	All experts gave a rating of “2”
	**Color saturation** Color saturation refers to the density of color within the art product varying between transparent and impasto.	**Color saturation** The extent to which a color is saturated. The more saturated the color is, the less paper/canvas, etc., is apparent. **Texture** Texture refers to the tactile surface of the artwork. This does not depend on the paper/canvas, etc., used, but can depend on the art material used and the way it is applied.	All experts gave a rating of “1”

**FIGURE 5:**
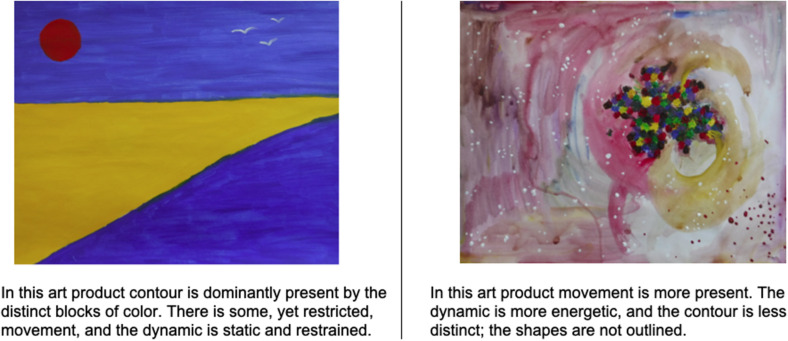
Examples of movement, dynamic, and contour of art products.

In summary, it was found that “movement,” “dynamic,” “contour,” and “mixture of color” were similar. These formal elements could be operationalized reliably and appeared to also be clinically relevant. See Figure 5 for examples of these formal elements in art products. A fourth study was then conducted to see whether these formal elements are related to mental health.

## Study 4: Relationship Between Formal Elements and Adult Mental Health

Art therapy literature demonstrates a wide range of opinions concerning the relationship between formal elements and mental health. In most studies, formal elements are related to disorders of the Diagnostic and Statistic Manual of Mental Disorders (DSM) or the International Statistical Classification of Diseases and Related Health Problems (ICD) (Gantt and Tabone, 1998; Hacking, 1999; Kim et al., 2012). Whereas in more recent studies formal elements are related to clients’ strengths (Hinz, 2009, 2015; Pénzes et al., 2014, 2015) in line with perspectives on positive mental health (Huber et al., 2016). Thus, literature has been inconsistent in interpreting formal elements of art product in terms of mental health.

According to our previous study, art therapists considered mental health in terms of balance and adaptability. Balance existed when allowing, experiencing and expressing emotions and cognitive control were in proportion to each other. Adaptability consisted of self-management, flexibility, openness, and creativity. Art therapists associated balance, specifically being out of balance, with the severity of clients’ problem and adaptability with clients’ strengths and resources. The ability to assess the level of balance and adaptability may support art therapists to formulate treatment goals to suit the individual needs and potential and to choose art interventions that enhance or develop the client’s mental health (Pénzes et al., 2018). Also, in our previous study we showed that art therapists did not related individual formal elements to mental health; they related specific *combinations* of formal elements, specifically the primary formal elements of “dynamic,” “movement,” and “contour” to mental health.

In this study we tested the combination of formal elements and the relation to mental health.

### Method

Forty-six adult clients in nine different mental health settings participated in this study. These were the same clients and art products examined in the final round of study 2. Client inclusion criteria included being aged between 18 and 65 years, being able to read and speak Dutch, and having no or minimal experience in making art and art therapy. Clients with psychotic disorders or in manic episodes were excluded. Art products were made with standardized instructions (see the final round in study 2). The two raters in the final round in study 2 independently rated the formal elements. Written informed consent was obtained from all participants in accordance with the Declaration of Helsinki.

The sampled clients (34 female, 12 male) started art therapy in nine different settings for adult mental health care ranging in age from 19 to 60 (*M* = 37). They all just started art therapy (<3 weeks), just after a psychologist gave a preliminary DSM-diagnosis. The sample was heterogenic regarding clinical diagnoses, 18 clients had a dual-diagnosis, nine clients had triple-diagnoses, and three clients four or more diagnoses. Most frequently diagnosed DSM categories were personality disorder (31 clients, 67%) and mood disorder (27 clients, 62%).

During the same week that the client made their art product, they filled in the Dutch version of the Brief Symptom Inventory (BSI) (de Beurs and Zitman, 2006), the Resilience Scale (RS-nl) (Portzky et al., 2010), and the Acceptance and Action Questionnaire – II (AAQ-II) (Jacobs et al., 2008). The BSI was used to assess the level of the clients’ mental illness/psychopathology, while the RS-nl and the AAQ-II were used to assess the clients’ mental health.

The BSI was a self-rating instrument consisting of 53 items derived from the SCL-90 and is used for the screening and assessment of psychopathology. The BSI was considered appropriate for patients without severe psychiatric disorders. Psychometric qualities were in line with the SCL-90 (alpha > 0.71) with good correlations with other instruments (Buwalda et al., 2012). The respondents scored the extent to which they experienced diverse psychological symptoms during the last week on a five-point Likert scale ranging from “a lot” to “not at all.” The BSI consists of three scales and nine subscales, including a general measure of psychopathology (total score). In this study, only the total score of the BSI was used because the art therapists described mental illness in general terms instead of specific disorders or symptoms.

The RS-nl was a self-rating instrument for screening resiliency. The respondents scored the extent to which they agree with 25 items on a four-point Likert scale ranging from “totally agree” to “totally disagree.” The RS-nl consists of two subscales: “personal competency” and “acceptance of self and live.” A high total score suggests adaptability, balance, and flexibility. Psychometric qualities are “good” for content and construct validity and for internal consistency (Windle et al., 2011).

The AAQ-II measured the amount of experiential acceptance, experiential avoidance, and psychological (in)flexibility. It consisted of 10 items that respondents scored using a seven-point Likert scale ranging from “never true” to “always true” (Bond et al., 2011). A high total score indicated higher experiential acceptance or psychological flexibility and less experiential avoidance. Persons that scored high on experiential avoidance generally used avoidance strategies that were related to repression of negative emotions and higher psychopathology. Psychometric qualities showed good internal consistency (Cronbach Alpha = 0.89) and construct validity of −0.67 to −0.79 on repression and psychological complaints (Jacobs et al., 2008; Fledderus et al., 2012; Bernaerts et al., 2014).

As art therapists related the combination of formal elements to mental health, Spearman correlation analysis was used to test whether the formal elements of “movement,” “dynamic,” and “contour” were related to each other. Linear regression analysis was used to test whether the combination of the formal elements “movement,” “dynamic,” and “contour” was related to clients’ scores on the BSI, RS-nl, and AAQ-II. All analyses were conducted in SPSS version 25.

### Results

Table 4 presented an overview of the mean scores, standard deviations, and variances for the formal elements “movement,” “dynamic” and “contour” and for the BSI, RS-nl, and AAQ-II scores.

**TABLE 4:** Descriptive statistics for the formal elements dynamic, movement and contour, and the BSI, RS-nl, and AAQ-II scores.

	Mean	Std. Deviation	Min.	Max.
Dynamic	2.50	0.66	1	4
Movement	3.00	0.82	2	4
Contour	2.50	0.78	1	4
BSI	90.07	36.45	12	163
RS-nl	60.77	10.25	40	80
AAQ-II	30.60	9.18	17	51

#### Inter-Relatedness of Formal Elements

Results of the Spearman correlation analysis indicated a positive significant relationship between “movement” and “dynamic:” *r*_*s*_ (46) = 0.76, *p* < 0.001. This suggests that an increase in “movement” is related to an increase in “dynamic” and vice versa. The results also indicated a significant negative relationship between both “movement” and “contour:” *r*_*s*_ (46) = −0.41, *p* = 0.004 and “dynamic” *r*_*s*_ (46) = −0.34, *p* = 0.020 and “contour.”

#### Relationship Between Formal Elements and Mental Health

Results of the regression analysis (see Table 5) showed that even though “dynamic” and “movement” significantly related to the BSI total score, the overall model was not significant (*R*^2^ = 0.16, *p* = 0.072). The overall model significantly related to the AAQ-II total score (*R*^2^ = 0.29, *p* = 0.003), however, with main effects found for “dynamic” (β = 0.60, *p* = 0.004) and “movement” (β = −0.76, *p* < 0.001). The overall model also significantly related to the RS-nl total score (*R*^2^ = 0.19, *p* = 0.034) with one main effect found for “dynamic” (β = 0.65, *p* = 0.004) and a trend for “movement” (β = −0.40, *p* = 0.070). The combination of “movement,” “dynamic,” and “contour” explained 19.3% of variance in RS-nl scores and 29.1% of the variance in AAQ-II scores. The combination of these formal elements was thus significantly related to resiliency and to psychological flexibility/experiential acceptance but not to the level of complaints.

**TABLE 5:** Regression analysis results: relationships between the formal elements dynamic, movement and contour and the BSI, AAQ-II, and RS-nl total scores.

	BSI total score		AAQ-II		RS-nl	
	B (SE)	B	B (SE)	β	B (SE)	β
Dynamic	−30,494 (34,777)	−0.556*	8,350 (2,741)	0.602**	9,977 (3.271)	0.647**
Movement	20,785 (9,834)	0.459*	−8,647 (2,247)	−0.764***	−5,040 (2, 709)	−0.396
Contour	−7,109 (7,228)	−0.154	1,246 (1,669)	0.107	1,712 (1,991)	0.132

B, Unstandardized B; SE, Standard Error; β, Beta coefficient. *p < 0.05, **p < 0.01, ***p < 0.001.

## Discussion

In study 1 seventeen formal elements emerged from the analysis of art theories. Findings of study 2 show that the majority of these seventeen formal elements can be observed reliably. Based on the results in Study 3, the formal elements “dynamic,” “movement,” and “contour” are found to be primarily clinically relevant and can be measured reliably. Results of Study 4 show that the combination of the formal elements “dynamic,” “movement,” and “contour” explain 19% of the variance in RS-nl scores and 29% of the variance in AAQ-II scores. These findings imply that the combination of the formal elements “dynamic,” “movement,” and “contour” are related to adult clients’ resiliency and experiential acceptance. The same combination of elements shows only a slight non-significant statistical relation to the BSI; even though “dynamic” and “movement” related significantly tot the BSO total score, the overall model that also included “contour” is not significantly related. Looking at each formal element, “movement” and “dynamic” are significantly related to clients’ mental health, whereas “contour” is not. The relationship is most pronounced for positive mental health concepts, such as experiential acceptance and resiliency. These results are in line with other art therapy studies that stress the importance of positive mental health in art therapy assessment (Hinz, 2009; Betts, 2012; Pénzes et al., 2018). Rather than focusing on DSM diagnoses or symptoms, art therapists often emphasize clients’ strengths and resources, embracing a perspective of mental health that is in line with the idea of positive health (Huber et al., 2016) and the recovery approach (Anthony, 1993; Jacob et al., 2017). These results are somewhat unexpected as Haeyen et al. (2017) found the same effect sizes for flexibility and mental illness (OQ-45 and SMI maladaptive scales) in their study on effects of art therapy for patients with a personality disorder. A variety of hypotheses can be formulated to account for these outcomes: maybe the differences are related to differences in patient samples, the use of specific measures (BSI versus OQ-45 and SMI), of differences in study design (we did not study the effects of an art therapy intervention and did not calculate effect sizes). In addition, a recent scoping review of Lasiello et al. (2020) identified a considerable body of empirical research investigating the validity of the dual-continua model, and the overarching notion that positive mental health and mental illness represent two distinct, yet related, constructs. They strongly advocate assessing positive mental health and mental illness together, rather than using only one or the other. Our results are in line with their conclusions; a focus on either positive mental health or mental illness alone would not provide a complete image of the mental health status of an individual or population. Undoubtedly, further research is needed to rule out the different hypotheses and to enlighten us in more detail about the nature of the relation between illness and positive health.

The results of Study 4 also indicate that the *combination* of formal elements is related to mental health and that the involved formal elements are strongly interrelated, e.g., an increased presence of “dynamic” and “movement” is related to a decreased presence of “contour.” This is in line with existing studies that have argued that individual elements mean nothing unless considered as a cluster (Gantt, 2001) or specific combinations of “primary” formal elements that construct the “structure” of the art product (Pénzes et al., 2018).

“Movement” and “dynamic” are strongly interrelated, indicating that an increase in “movement” is related to an increase in “dynamic.” When relating these elements to mental health measures, however, “movement” is negatively associated with mental health and “dynamic” is positively related. A possible explanation for this can be found in a previous study in which the concept of “variation” emerged (Pénzes et al., 2018). “Variation” refers to the diversity that can be recognized in one or more formal elements within the art product. Art products with low “dynamic” and low “movement” or high “dynamic” and high “movement” have less “variation.” In contrast, art products with low “dynamic” and high “movement” or vice versa have more “variation.” Art therapists largely agree on the importance of observing this aspect of art products to assess client mental *health.* Future research could address operationalizing “variation” to investigate its relationship with mental health.

In summary, the relationships between formal elements, positive mental health, and mental illness show that art products are indeed associated with aspects of positive mental health and mental illness. The classic assumption of art therapists is in line with this. This implies that the lack of reliability and validity of existing art therapy assessment instruments is not due to the absence of such a relationship or to incorrect theoretical assumptions, but to inconsistent operationalization or weak study design.

### Implications for Clinical Practice of Art Therapy

Despite the ongoing discussion about how to interpret the formal elements in art therapy assessment (e.g., Hinz, 2009; Gilroy et al., 2012), most studies seem to agree that formal elements are related to *how* clients make art products (Gantt, 2001; Hinz, 2009; Conrad et al., 2011). In previous studies, this assumption has been specified using the concept of “material interaction,” which refers to the art making process and the clients’ interaction with the art materials (Pénzes et al., 2014, 2015). In these studies, it is conceptualized that material interaction is reflected by the formal elements of the art product and related to mental health. The significance of the combination of “movement” and “dynamic” might imply that these formal elements reflect the clients’ art making, i.e., the amount and kind of movements that clients make when interacting with art materials to create an art product.

The “dynamic” and “movement” of the art products and “movement” of material interaction can be linked to Stern’s (2010) concept of “forms of vitality,” which refers to the forces and sensations linked to the movement that creates forms. Stern states that peoples’ health can be evaluated based on the vitality that is expressed in their almost constant movements. This implies that formal elements might be considered as manifest visual forms of vitality and that these formal elements in art products reflect mental health and mental illness.

Further research is needed to investigate the possible relationship between “dynamic” and “movement” in clients’ art products and clients’ “material interaction” in art making. For the time being, these two formal elements are observable and visible aspects of the art product. This visibility allows the joint reflection of the art therapist and the client, leading to insights and awareness that may help formulate treatment objectives and art interventions. The relationship between the formal elements and mental health is most pronounced for positive mental health aspects, such as experiential acceptance, adaptability, and resiliency. This may have implications for art therapy assessment and treatment. The clients’ adaptability, flexibility, and resiliency may indicate the potential for change in therapy and art therapists in a previous study associated adaptability with clients’ prognoses in therapy (Pénzes et al., 2018). Several studies have also shown the therapeutic potential of diverse art material properties to create experiential interventions that allow change and ameliorate mental health (Hinz, 2009; Moon, 2010; Snir and Regev, 2013; Pénzes et al., 2014). This assumption requires further longitudinal research.

### Methodological Considerations

In this study, art products made with diverse materials and instructions are included. The analyses of studies 3 and 4 are based on art products made with acrylic paint. This is a serious limitation of our study as the art materials and instructions may have influenced the results. For example, the formal element “filled space” might have emerged when art products were made on larger pieces of paper, with smaller brushes, and/or with more time allowed.

We excluded “mixture of color” from the analysis in study 4 because theoretically, it was not conceptualized as a “primary” formal element. The primary formal element “repetition” was also excluded from analysis, as it did not show enough resemblance to formal elements in art theories. Future research could include these formal elements to investigate if they influence the formal elements’ relationship with mental health measures.

The relationship between the formal elements and mental health found in this work is based on a cross-sectional study design, which does not allow for insights into causality and effects over time. Longitudinal studies including a series of art products could investigate whether therapeutic experiential art interventions change the art making, i.e., material interaction, and whether these are reflected in the formal elements of “movement” and “dynamic” and in the enhancement of experiential acceptance, adaptability, and resiliency.

In study 3 we compared formal elements from art theory to formal elements that art therapists in a previous study found relevant in clinical practice. The majority of these art therapists practice in the Netherlands. Although we did include a few art therapists from other countries such as Israel, Finland, Germany, the United States, and United Kingdom to contrast findings, further generalizability of art therapists’ perspectives may need to be checked through further research.

## Conclusion

The combination of the formal elements “movement,” “dynamic,” and “contour” are significantly interrelated and related to clients’ mental health, i.e., psychopathology, psychological flexibility, experiential avoidance, and adaptability. Numerous questions remain unanswered and require further research. Even though it is methodologically challenging to study art and the art-making process in relation to mental health, this study highlights the possibilities for future research. Art products seem to have diagnostic value and may add to clients’ verbal expression and indicate their potential to benefit from therapy.

## Data Availability Statement

The raw data supporting the conclusions of this article will be made available by the authors, without undue reservation.

## Ethics Statement

Ethical review and approval was not required for the study on human participants in accordance with the local legislation and institutional requirements. The patients/participants provided their written informed consent to participate in this study.

## Author Contributions

IP developed the research design, conducted the research, and first authored this article. SH, DD, and GH supervised the development of the research design and research process, and co-authored this article. All authors contributed to the article and approved the submitted version.

## Conflict of Interest

The authors declare that the research was conducted in the absence of any commercial or financial relationships that could be construed as a potential conflict of interest.
